# Fasting: a major limitation for resistance exercise training effects in rodents

**DOI:** 10.1590/1414-431X20175427

**Published:** 2017-11-17

**Authors:** W. das Neves, L.F. de Oliveira, R.P. da Silva, C.R.R. Alves, A.H. Lancha

**Affiliations:** Escola de Educação Física e Esporte, Universidade de São Paulo, São Paulo, SP, Brasil

**Keywords:** Strength, Physical activity, Hypertrophy, Atrophy, Skeletal muscle wasting

## Abstract

Protocols that mimic resistance exercise training (RET) in rodents present several limitations, one of them being the electrical stimulus, which is beyond the physiological context observed in humans. Recently, our group developed a conditioning system device that does not use electric shock to stimulate rats, but includes fasting periods before each RET session. The current study was designed to test whether cumulative fasting periods have some influence on skeletal muscle mass and function. Three sets of male Wistar rats were used in the current study. The first set of rats was submitted to a RET protocol without food restriction. However, rats were not able to perform exercise properly. The second and third sets were then randomly assigned into three experimental groups: 1) untrained control rats, 2) untrained rats submitted to fasting periods, and 3) rats submitted to RET including fasting periods before each RET session. While the second set of rats performed a short RET protocol (i.e., an adaptation protocol for 3 weeks), the third set of rats performed a longer RET protocol including overload (i.e., 8 weeks). After the short-term protocol, cumulative fasting periods promoted loss of weight (P<0.001). After the longer RET protocol, no difference was observed for body mass, extensor digitorum longus (EDL) morphology or skeletal muscle function (P>0.05 for all). Despite no effects on EDL mass, soleus muscle displayed significant atrophy in the fasting experimental groups (P<0.01). Altogether, these data indicate that fasting is a major limitation for RET in rats.

## Introduction

Resistance exercise training (RET; also known as strength training) is a well-established non-pharmacological strategy to promote skeletal muscle hypertrophy and functionality in humans. For decades, RET have been prescribed to 1) improve sports performance, 2) promote a healthy lifestyle, and 3) counteract aging effects and a large spectrum of chronic disease worldwide (1). However, the molecular basis underlying RET benefits is still unclear (2–4). Animal studies have been useful to explore deeper mechanisms underlying skeletal muscle remodeling, including the effects of physical exercise. While aerobic exercise training protocols could mimic most human regimens (considering the differences between species), there is a lack of a robust RET protocol for rodents. Most RET protocols for rodents present limitations, compromising data extrapolation to humans (5).

Tamaki et al. (6) proposed a RET for hindlimb (i.e., “squat-like” movement) in which rats were suspended in a standing position and fixed into an apparatus using a canvas jacket. RET performance in this apparatus improves skeletal muscle strength and function. However, in order to perform squat movements, animals are electrically stimulated, which is beyond the physiological context observed in humans (7). Another typical resistance exercise for rodents is ladder climbing (4,8). Although no electrical stimuli are necessary for exercise execution, some concerns regarding ladder protocols exist, such as the high aerobic demand leading to cardiac hypertrophy mischaracterizing the protocol as a “pure” RET, and observations that minor modifications to the ladder protocols may greatly affect the results, which has not created a consensus regarding the real efficacy to induce skeletal muscle hypertrophy (5,9).

In this sense, our group developed an operant conditioning system composed of sound, light and feeding devices that does not use electric shock as stimuli for resistance exercise execution and allows a good control of RET variables such as the rest interval between sets and repetitions. Additionally, even though this protocol is based on sound/light and food reward, we have reported that theoretically this system does not impose fasting periods (2). Unfortunately, we now found that without a fasting period, the training process is not sustainable. Considering this limitation, the current study was designed to test whether a long fasting period before each RET session has some influence on skeletal muscle adaptation. For that, we evaluated the effects of consecutive fasting periods in untrained rats and rats submitted to both short and long RET protocols adapted from a pioneer study (2).

## Material and Methods

### Sample and experimental design

Male Wistar rats (12-weeks old, 300–400 g) were housed (3 or 5 per cage) in an animal facility under controlled temperature (21°C) with an inverted 12:12 h light:dark cycle. Animals had access to standard laboratory chow and water. All experiments were conducted during the dark period in a dark room because rodents have nocturnal habits. Rats were divided in three sets for three independent experiments. The first set of rats (n=6) was used for a pilot experiment (experiment 1). In this experiment, all animals were submitted to the RET protocol previously proposed (2) without food restriction. Based on the results of this pilot experiment, two new sets of rats were used. The second set (18 animals) was randomly assigned into three experimental groups: 1) untrained control rats (control; n=6), 2) untrained rats submitted to cumulative fasting periods (fasting; n=6), and 3) rats submitted to a short RET adaptation protocol with fasting periods (fasting + RET; n=6). The follow-up time lasted 3 weeks and food intake, body mass and extensor digitorum longus (EDL) mass were assessed in this first set of rats. The third set (15 animals) was randomly assigned into three different experimental groups: 1) untrained control rats (control; n=5), 2) untrained rats submitted to fasting periods (fasting; n=5), and 3) rats submitted to a longer RET protocol with fasting periods (fasting + RET; n=5). The follow-up time lasted 8 weeks and food intake, body mass, EDL and soleus mass, EDL fiber cross sectional area and *in vivo* skeletal muscle function were assessed in this third set of rats.

The current study was approved by the local Ethical Committee (Escola de Educação Física e Esporte, Universidade de São Paulo; protocol: 2013/02). All procedures were performed in accordance with the Guide for the Care and Use of Laboratory Animals (National Institutes of Health, USA) and with ethical principles in animal research adapted by the Brazilian College of Animal Experimentation (www.cobea.org.br).

### RET and fasting protocols

RET for hindlimb (i.e., “squat-like” movement) was performed using an apparatus and protocol adapted from Nicastro et al. (2). As shown in Table 1, the protocol consists of different phases: Magazine, Nose poke 1, Nose poke 2, Standing 1, Standing 2, Lifting 1 and Lifting 2. The different phases induce a progressive stimulus and adaptation for the resistance exercise performance. Each RET session was interposed by 48 h (during the weekdays) or 72 h (during the weekend) resting periods. Importantly, rats were submitted to a 24-h fasting period before each RET session, but standard laboratory chow was provided *ad libitum* after each RET session. Moreover, the overload applied during the “Lifiting 2” phase was based on the body mass of each animal. Animals were weighed weekly and load adjustments were applied accordingly.

**Table 1.** Resistance exercise training (RET) protocols applied in the first, second and third sets of rats.

Phase	No. of RET sessions	No. of repetitions/session	Overload (g)
**First and second sets of rats: short RET adaptation protocol (3 weeks)**			
Magazine	1	30	0
Nose poke 1	1	30	0
Nose poke 2	2	30	0
Standing 1	2	30	0
Standing 2	2	30	0
Lifting	1	30	0
Lifting	1	30	50% body weight
**Third set of rats: long RET protocol (8 weeks)**			
Magazine	1	30	0
Nose poke 1	1	30	0
Nose poke 2	2	30	0
Standing 1	2	30	0
Standing 2	2	30	0
Lifting	2	30	0
Lifting	14	30	50% body weight

### Body mass, food intake and skeletal muscle morphology

Body mass was assessed using a small animal weighing scale (Gehaka Ltda., Brazil); food intake was assessed in each cage (3 rats per cage). To measure EDL and soleus mass, rats were anesthetized with isoflurane and killed by decapitation. Left EDL and soleus muscle were carefully harvested, weighted and frozen. To provide a relative comparison for each muscle mass, data were normalized by body mass. Frozen EDL muscles were cut into 10-µm cross-sections using a cryostat (Micron HM505E, Zeiss, Germany) at –20°C. Sections were submitted to eosin-hematoxylin staining. Cross-sectional area was evaluated in >200 EDL fibers at 200× magnification and further analyzed on a digitizing unit connected to a computer (Image-Pro Plus, Media Cybernetics, USA). Analyses were performed by an investigator who was blind to the rat identities.

EDL and soleus muscles were chosen due to the high prevalence of type II and type I fibers, respectively. There are large differences between type I and type II fibers regarding their speed of contraction, oxidative capacity and fatigue resistance. Additionally, different fiber types are known to display different responses to metabolic stimuli and exercise (10,11). In fact, loss of mass was not found in soleus muscle after different caloric restriction protocols, while other muscles displayed atrophy (12,13). Additionally, most of the hindlimb muscles are recruited during the “squat-like” movement and EDL and soleus seem to adapt after different RET protocols (9,14,15).

### Skeletal muscle function

In the third set of rats, *in vivo* skeletal muscle function was estimated by both rotarod and ambulation tests before and after the RET protocol. Rotarod test determined the time that each rat was able to stay on a gyratory rod. The equipment (AVS, Brazil) was programmed to present an initial speed of 1 rpm and a final speed of 40 rpm, 300 s later. The ambulation test (also known as footprint test) determined the mean length of a step, measured in hind foot ink prints while rats ran freely in a corridor. Rats were subjected to three successive trials for both rotarod and ambulation tests and the averaged values were considered for statistical analysis (16).

### Statistical analysis

Data are reported as means±SE or percentage change from control group, when appropriated. Analyses were conducted using Graph Pad Prism 6 (Graph Pad Software Inc., USA). All variables were tested for outliers using Grubbs' test and no significant outlier value was found. One-way ANOVA was applied. Whenever significant effects were observed, a Fisher's least significance difference test was used for multiple comparison purposes. Statistical significance was set at P<0.05.

## Results

### Experiment 1: pilot experiment without food restriction

Data from this pilot experiment are reported in Supplementary Table S1. Only 2 from a total of 6 rats were able to perform voluntary “squat-like” movements during the adaptation phases of the RET protocol, while no animals were able to perform the lifting phase.

### Experiment 2: short RET protocol with fasting periods before each RET session

**Figure 1.**
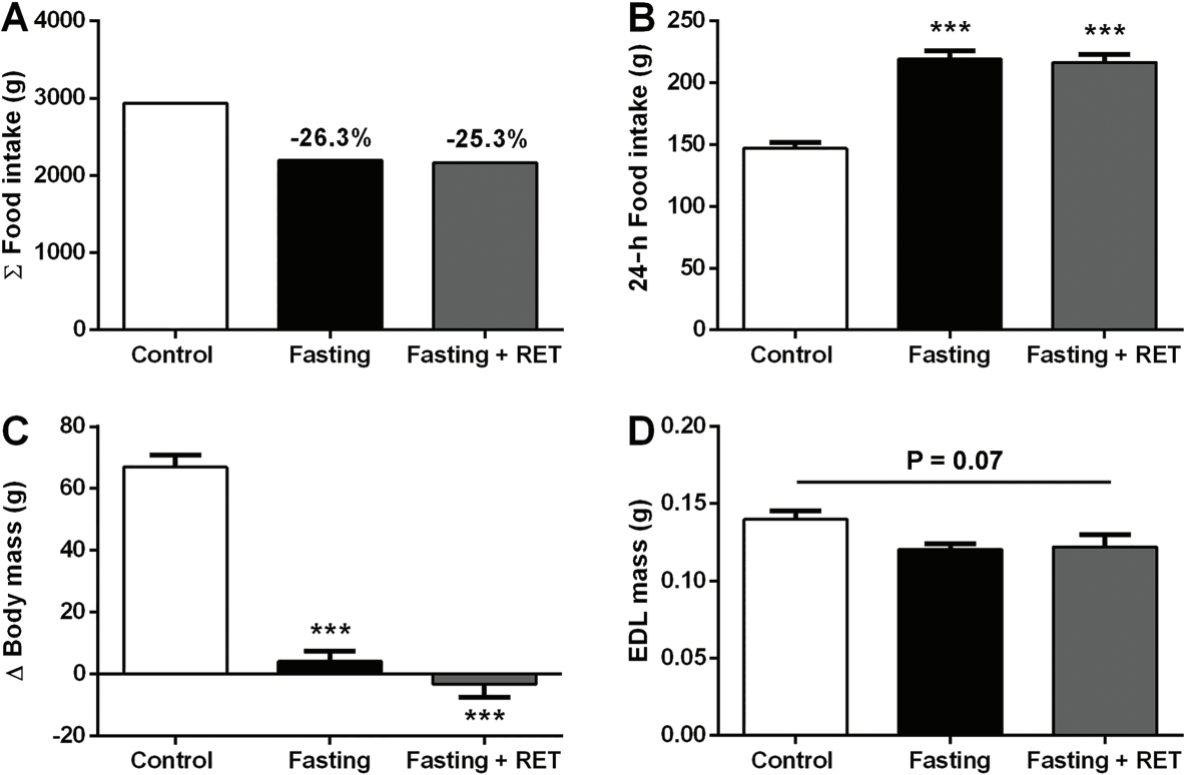
Effects of fasting during the short resistance exercise training (RET) adaptation protocol (first set of rats). Total food intake after the 3 weeks follow up (*A*), 24 h food intake immediately after each fasting period (*B*), changes of body mass (*C*) and extensor digitorum longus (EDL) muscle mass (*D*) for control, fasting and fasting + RET experimental groups. Data are reported as means and percentage changes from control group (*panel A*) or means±SE (*panels B* to *D*). ***P<0.001 compared to the control group (n=6) (ANOVA and Fisher’s least significance difference test).

As shown in Table 1, the first set of rats performed a RET adaptation protocol with a total duration of approximately 3 weeks. Fasting (–26.3%) and fasting + RET (–25.3%) groups reduced total food intake (Figure 1A), although they had a compensatory food consumption after the food restriction period (Figure 1B; P<0.001). This reduction in the total food intake resulted in a severe weight loss (Figure 1C; P<0.001). No differences were observed in EDL mass (Figure 1D; P=0.07) or EDL mass normalized by body mass (P>0.05; data not shown) among experimental groups. Importantly, there was no significant difference between fasting and fasting + RET groups for any variable (Figure 1A–D; P>0.05 for all variables).

### Experiment 3: long RET protocol with fasting periods before each RET session

**Figure 2.**
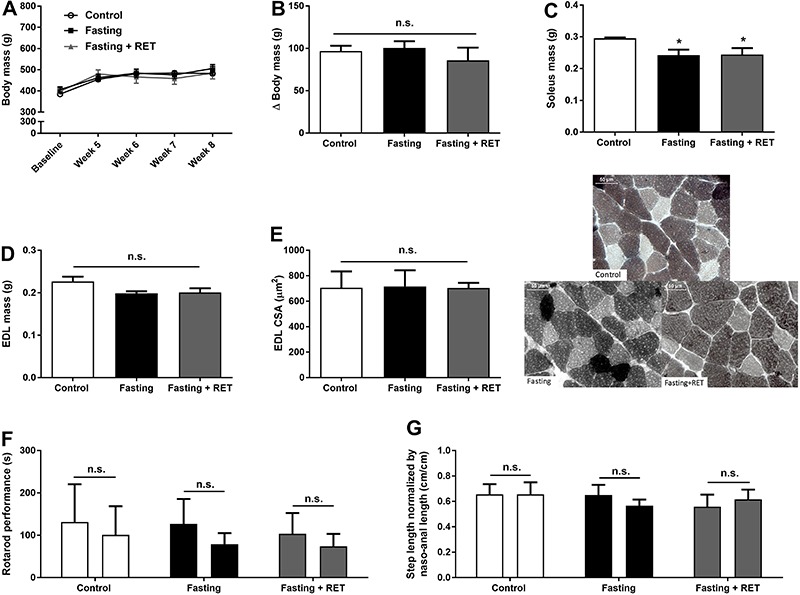
Effects of fasting during the long resistance exercise training (RET) protocol (second set of rats). Time-course of body mass (*A*), changes of body mass (*B*), soleus muscle mass (*C*), extensor digitorum longus (EDL) muscle mass (*D*), EDL fiber cross-sectional area (CSA) and corresponding microphotograph (*E*), rotarod test performance pre- and post-RET protocol (*F*), and ambulation test performance pre- and post-RET protocol (*G*) for control, fasting and fasting + RET experimental groups. Data are reported as means±SE (n=5). *P<0.05 compared to the control group. n.s. indicates non-significant differences (ANOVA).

In the second set of rats, a longer RET protocol was conducted (Table 1). No difference among experimental groups was observed in body mass (Figure 2A and B; P>0.05). Soleus displayed significant atrophy in the fasting group (Figure 2C; P<0.01), while no difference was found in EDL mass (Figure 2D; P>0.05). Similar results were observed when EDL and soleus mass were normalized by body mass (data not shown). EDL morphology was confirmed by histological analysis, showing no difference among experimental groups in fiber cross-sectional area (Figure 2E; P>0.05). No difference was found for rotarod and ambulation performance, suggesting that *in vivo* skeletal muscle function was unchanged. Finally, there was no significant difference between fasting and fasting + RET groups for any variable (Figure 2A-G; P>0.05 for all variables).

## Discussion

Our group has developed an operant conditioning system composed of sound, light and feeding devices that does not use electric shock as stimuli for resistance exercise execution and allows a good control of RET variables. Additionally, previous data suggested that fasting is not necessary to stimulate exercise in this system (for details, please see Ref. 2). However, we now found that without fasting the exercise training program is not sustainable. Non-fasting animals actually do not perform the exercise even after the adaptation period. Thus, the current study aimed to evaluate whether consecutive long fasting periods have some influence on skeletal muscle adaptation after a short and long RET protocols in rats.

The main findings indicate that animals presented loss of weight immediately after a short RET adaptation protocol (i.e., 3 weeks) as consequence of the cumulative fasting periods. On the other hand, no differences were observed in the body mass, and muscle morphology or functionality after a longer RET protocol (i.e., 8 weeks), suggesting that some compensatory response mitigated the fasting effects. Despite no difference in EDL morphology, the soleus muscle displayed atrophy after the long RET protocol. The soleus muscle has a high prevalence of type I fibers (i.e., oxidative fibers), which display lower response to aging, cachectic and RET stimuli (10,11,17). Moreover, loss of mass was not found in soleus muscle after different caloric restriction protocols (12,13). To our knowledge, this is the first study that assessed interposed and cumulative long fasting periods in muscles with different phenotypes. As type II fibers are highly responsive to RET stimuli, it is possible that muscle atrophy induced by fasting was restored by RET stimuli in the EDL muscle, which was not observed in the soleus muscle with prevalence of type I fibers. Unfortunately, the lack of an experimental group performing RET without food restriction does not allow confirming this hypothesis.

The maximal volitional strength capacity can be defined as the maximal load with which rats are able to perform a single squat by using the current operant system (2,15). However, it is expected that a model employing starvation will motivate the animal to perform a task involving only 50–60% of its maximal voluntary capacity (5). In fact, by using RET protocols based on electrical stimulus, animals could squat with loads 2.5-fold greater than in the current protocol (2,7). Therefore, here we decided to include other functional tests to assess skeletal muscle function *in vivo*. Two well-established tests (i.e., rotarod and ambulation) were applied (16). However, no benefit of RET was observed in either test performance. Altogether, these data demonstrated that the current RET protocol was ineffective to promote any effect in skeletal muscle mass and function.

This study is not without limitations. First, we did not test the effects of shorter periods of fasting (e.g., 12, 6, or 3 h) before each RET section. It could be speculated that a short fasting period does not culminate in an unwanted effect on muscle mass, however we are not sure if a short fasting period could stimulate exercise execution. We performed a 24 h fasting period to ensure stimulus for the exercise execution, but further experiments should test the impact of shorter fasting periods in the body/muscle mass as well as exercise viability. Second, in this study animals were maintained in social groups to avoid any other variable that could change animal behavior. Therefore, food intake was assessed in each cage with three animals per cage. Although other studies have used this type of measurement per cage (18–20), we cannot ensure that rats equally reduced their food intake. Finally, in this study we focused on the chronic effects of RET and we did not assess the fasting effects after a single bout of exercise. The lack of acute measurements is an additional limitation of the current study.

In summary, this study demonstrated that cumulative long fasting periods were a major limitation for RET in rats. The lack of a robust RET protocol for rodents compromises the extrapolation of any data to humans. The development of new approaches to mimic resistance exercise in rodents is still necessary to investigate the mechanisms underlying skeletal muscle remodeling.

## Supplementary material

Click here to view [pdf].
